# Environment and child well-being: A scoping review of reviews to guide policies

**DOI:** 10.34172/hpp.2023.20

**Published:** 2023-09-11

**Authors:** Louise Wallerich, Amandine Fillol, Ana Rivadeneyra, Stéphanie Vandentorren, Jérôme Wittwer, Linda Cambon

**Affiliations:** ^1^University of Bordeaux, INSERM, BPH, U1219, Mérisp/PHARES, Equipe Labellisée Ligue Contre le Cancer, CIC 1401, F-33000 Bordeaux, France; ^2^Institute of Public Health, Epidemiology and Development, Bordeaux, France; ^3^Equipe PHARes, Bordeaux Population Health, Bordeaux, France; ^4^French Public Health Agency, F-94415 Saint-Maurice, France; ^5^University of Bordeaux, Centre INSERM U1219 Bordeaux Population Health Research Center, Bordeaux, France

**Keywords:** Child, Child health, Environment, Review, Social determinants of health

## Abstract

**Background:**

Acting on social determinants is the most effective, efficient, and fairest strategy to improve population health and health equity. Because of their vulnerability and dependence, children are particularly exposed to the deleterious effects of their living environment. Taking these issues into account in the development of public policies and identifying levers for action are crucial. The objective of this scoping review of reviews is to identify the main environmental determinants on children’s health and development, and their mechanisms of effect, to be addressed by public policies.

**Methods:**

We conducted a scoping review of reviews in accordance with the method developed by Arksey and O’Malley, and Levac and colleagues’ methodology advancement and the PRISMA guideline. Inclusion criteria were identified with the PICos (population-phenomena of interest-context-study design) framework. We used the PubMed database and conducted a thematic analyze.

**Results:**

Forty-seven articles were selected. Their analysis allowed us to identify five categories of interdependent environmental determinants of child health: i) urban design ii) contaminants, iii) parenting environment, iv) social conditions, v) climate change. Together and in a systemic way, they act on the health of the child.

**Conclusion:**

The review carried out allows us to propose a pragmatic framework for clarifying the effects of the physical, social, and economic environment on children’s health and wellbeing.

## Introduction

 Childhood, defined as the period between prenatal development and 8 years of age, is recognized as the most crucial period of development in the human life span and the most highly sensitive to external influences.^1^ During this period, the foundations are laid for each individual’s physical, cognitive and mental capacities, influencing their subsequent growth, physical and mental health, and development throughout the life course.^2^ The social determinants of health (SDH) constitute the conditions under which people are born, grow, work, live, and age. They are shaped by the distribution of money, power, and resources, determine the conditions of daily life and tare responsible for most health inequities between and within countries.^3^ They are also the most powerful instruments to break the vicious circles of disadvantage at the start of life.^4,5^ The influence of SDH is all the more important for the youngest members of society, as they do not yet have the individual resources to cope with a detrimental environment or the ability to change this environment, as advocated in health promotion approaches.^6^

 Programs that aim to improve the environment in which children are born, grow up, live, and learn may therefore be instrumental in reducing health and developmental inequalities with lifelong effects on their life chances and subsequent health. That is why the World Health Organization, the United Nations, and the United Nations Children’s Fund are keen to promote a better understanding of the determinants of children’s health, and advocate for their wider inclusion in public policies.^7–9^ In particular, the need to act on children’s physical and socio-economic environments is clear.^10^ This is also strongly emphasized by the recent Geneva Charter,^11^ in line with the Health in All Policies framework.^12^ While the promotion of public health policies focused on the social determinants of children’s health is essential^13–17^ they are not always designed with this in mind.^18^ On the contrary, they tend to focus on behavioral aspects of child health (health education programs for parents and children) rather than on upstream social determinants (economic resources and living environments) that fundamentally shape behaviors, due in part to a lack of knowledge transfer between science and policy. In fact, public action should strive to ensure that the “world around the child” is favorable, i.e., containing “all the resources, infrastructures and networks that contribute to his or her well-being and development”.^19^ The present review is part of this pragmatic vision. Inspired by the Global Environmental Health Strategy,^20^ the theory of ecological systems^21^ and the multi-level framework of child well-being,^19^ it aimed at identifying and characterizing the environmental determinants of children well-being linked to the built and natural environment (housing, neighborhood, natural/green spaces, urban planning…) and to the socio-economic environment (social links, social capital, economic resources, family networks…) in which children are born, grow up, live, and learn. More precisely, this review aims to characterize the main physical and socio-environmental determinants of children’s health.

## Materials and Methods

###  Study design

 We conducted a scoping review of reviews in accordance with the method developed by Arksey and O’Malley,^22^ and Levac and colleagues’^23^ methodology advancement and the PRISMA guideline.^24,25^ According to Arksey and O’Malley, the scoping review approach was favored to “identify, retrieve and summarize literature relevant to a particular topic for the purpose of identifying the key concepts underpinning a research area and the main sources and types of evidence available.”^22^ More specifically, this review responds to the challenge described by Antman of summarising and disseminating research results. This type of scoping review can describe in more detail the results and scope of research in particular fields of study, thus providing a mechanism for summarising and disseminating research results to policy makers and stakeholders.^26^ Building on the scoping review approach, we chose to scope reviews rather than primary literature. The “scoping review of reviews” approach is efficient when it aims to map or explore of all available evidence around a given object of study. This type of scoping review has already been used by many authors.^27–30^ This type of review can describe in more detail the scope and results of existing research in particular areas of study, thus providing a means of summarizing research findings and disseminating them to policymakers. It was therefore particularly well-suited to our objective of proposing a conceptual framework the main social-environmental determinants to tackle by public policies.

 The steps for conducting a scoping review are as follows: (i) Identifying the research question, (ii) Identifying relevant studies, (iii) Study selection, (iv) Charting the data, (v) Collating, summarising and reporting the results.^22^

###  Defining the objectives and inclusion criteria

 The aim is (i) to characterize the socio-environmental determinants in the world around the child according the multi-level framework of child well-being^19^ and (ii) to propose a framework to guide the search for child-friendly solutions. The research questions were: What are the main environmental determinants influencing children’s physical and mental health and development? What are the mechanisms through which these determinants come into play? Inclusion criteria were identified with the PICos (population-phenomena of interest-context-study design) framework, as adapted of the PICO (population-intervention-comparison-outcomes) framework.^31^

 Such criteria included: The population of interest are children, defined as persons under 10 years old or elementary schoolchildren, without diagnosed conditions. The phenomena of interest are the physical environment (built or natural), social environment, economic environment, political environment, exposure, and climate environment related to children’s health. The study outcomes must be concerned with health/wellbeing or health behaviors. Furthermore, all types of health outcomes (physical, social, mental, or developmental) were considered in this review. No criteria were used to limit the context (geographical location of the studies or cultural factors). We included three study designs: reviews, systematic reviews, and meta-analysis as defined in the PubMed database. See Table 1.

**Table 1:** Inclusion and exclusion criteria

**Inclusion criteria**	**Exclusion criteria **
Population
Child (persons under 10 years old or elementary schoolchildren)	Children with diagnosed condition Adolescent, adult
Phenomena of interest
physical environment (built or natural), social environment, economic environment, political environment, exposure, and climate environment	Education, parenting, health prevention
Outcomes
health/wellbeing or health behaviors	
Design
Reviews, systematic reviews, and meta-analysis	Other study design
Period
2000-2023	Before 2000

###  Identification – Search strategy 

 We used the PubMed database. The algorithm was constructed from a goldset of seven articles previously identified in exploratory research^13,32–37^ and combining three approaches: (i) focusing on the social determinants of health; (ii) focusing on determinants related to physical and, socio-economic environments of children; (iii) focusing on child health and development. Mesh terms from the PubMed database were used for each approach. As “social determinants of health” is not a transdisciplinary term, it was necessary to add free terms and to broaden the search scope by targeting articles proposing or based on theoretical models referring to it. The strategy was limited by year of publication (between 2000 and 2022), by language (English or French), and by accessibility (full text).

 The study selection process consisted in three stages: (i) title screening, (ii) title and abstract screening, and (iii) full-text screening. The articles were selected based on the inclusion criteria by two researchers (AF, LW) using the *Covidence *toolkit. Disagreements were discussed until a consensus was reached.

###  Data extraction and analysis

 Data extraction was carried out once we had a final list of all the studies to be included in the review. A thematic analyze was conducted using the Nvivo^®^ software. The aim was to answer the following questions: What are the characteristics of the physical and/or socio-economic environments that influence the health of the child? What are their impact(s) and mechanisms of action? Two researchers (AF,LW) analyzed the data separately and then compared, combined and completed their analysis around the following elements: (i) description of the article: discipline and scope, type of article, theoretical approach, etc, (ii) types of children vulnerable to the determinant, (ii) mediators of the effect of the determinant or the mechanisms involved (defined as the reaction of an agent in a specific context^38^ which may be collective or individual), (iv) characteristics of the determinant, and (v) the health data.

## Results

###  Description 

####  Selection

**Figure 1:**
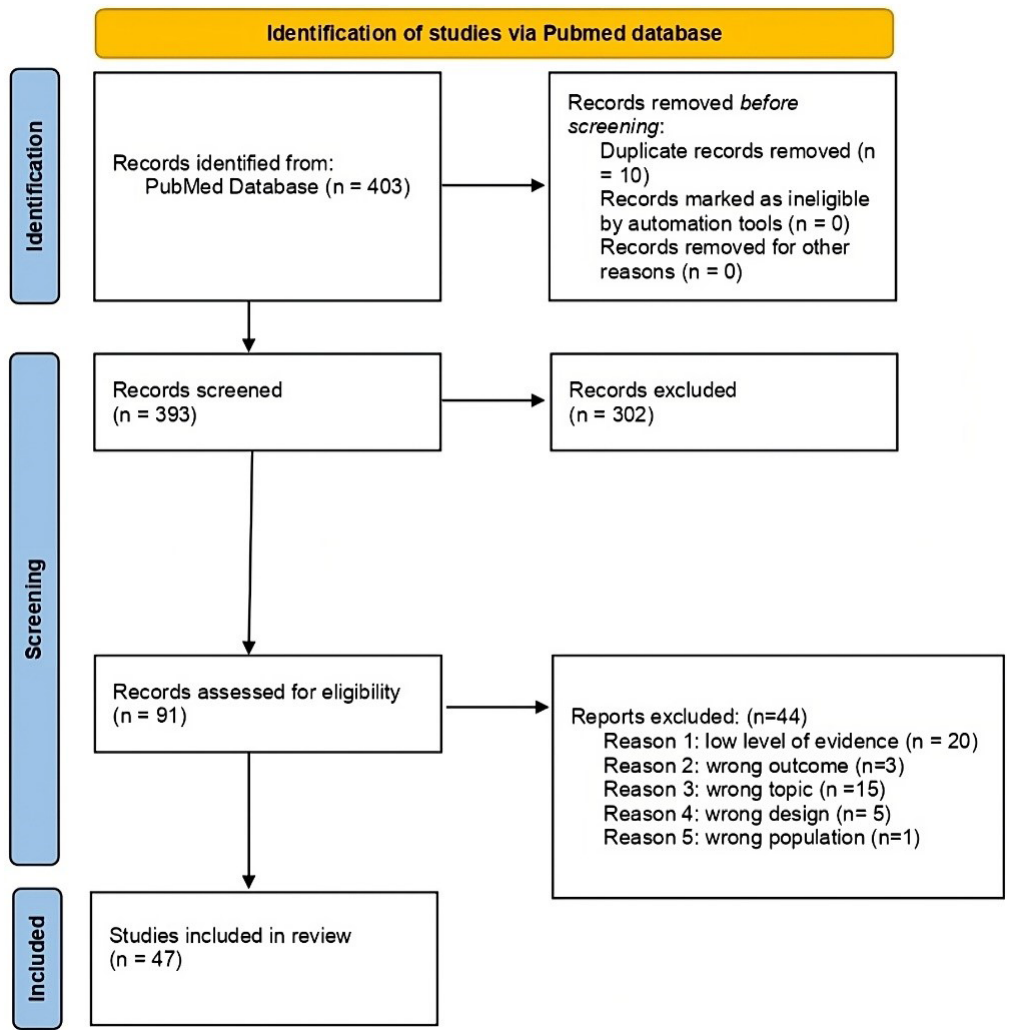
PRISMA flowchart of study selection process

 A total of 393 articles were identified after exclusion of duplicates. 91 articles were selected based on title and abstract. Of them, 47 articles were included in the review (Supplementary file 1),^2,13,29,37,39–80^ as shown in the flow chart in Figure 1.

 The articles excluded from the title and abstract screening did not meet one or more of the inclusion criteria: (*i*) articles that included the adolescent population; (*ii*) articles that did not fall within the defined “environment” section. In the full text screening, articles were excluded if they did not fit the intended design or did not examine any mechanism of action.

####  Description 

 Among the selected articles, 31 were reviews, 8 were systematic reviews, 3 were reviews of reviews, 2 were scoping reviews, 1 was a meta-analysis, 1 was an overview, and 1 was a research support.

 The authors’ disciplines were: Health Sciences (Medicine, Neuroscience, Pediatrics and Obstetrics, Gynecology and Reproductive Sciences), Public Health (Epidemiology, Health Equity, Environment and Health), and Humanities and Social Sciences (Anthropology, Science of education, social work, Psychology).

 More than half of the articles (25 out of 47) were published between 2018 and 2022.

 Eleven papers proposed theoretical models^29,32,39–47^ and 13 mentioned a multiplicity of determinants or interactions between several categories of determinants.^13,39,47–57^ The categories of determinants identified were: (i) urban planning: 6 articles,^32,37,46,58–60^ (ii) contaminants: 11 articles,^40,42,61–69^ (iii) the parenting environment: 2 articles,^70,71^ (iv) social conditions: 11 articles,^2,43–45,72–78^ and (v) climate change: 4 articles.^29,41,79,80^ A description of each paper is presented in Supplementary file 1.

 Among the health effects identified, we identified: (i) impacts on birth outcomes (preterm births, low birth weight, etc), (ii) impacts on the developmental process, with more or less long-term consequences, (iii) impacts on the prevalence of diseases throughout the life course, and (iv) children’s healthy behaviors.

###  The main environmental determinants of child health

 The results of the review identify 5 main categories of environmental determinants of child health and development: (i) urban design, (ii) contaminants, (iii) the parenting environment, (iv) socio-economic conditions, and (v) climate change. For each category, we have detailed, if it was possible, the causal pathways through which the most documented determinants can influence children’s health and development.

####  Urban planning and built environment

 The urbanization process is expected to continue in the coming years, reaching 60% of the world’s population by 2030.^81^ Urbanization and the shaping of the built environment have provided a number of socioeconomic benefits, but have also brought unwanted side effects, such as increasing exposure to air pollution, noise and high temperatures, less availability of or access to natural environments, and a more sedentary life.^82–85^ There is a growing interest in the interaction between urbanization and child health.^86^ In our review, 6 articles address this e subject through 3 dimensions: (i) access to green spaces, (ii) housing, and (iii) neighborhood organization.^32,37,46,58–60^

####  Access to green spaces 

 The analysis of the influence of green spaces on child development and health is relatively new. Green spaces include urban land covered with natural vegetation, such as parks, forests, wetlands and other ecosystems.^60^ To qualify these green spaces, several indicators are used, the most common being the Normalized Difference Vegetation Index (NDVI). According to Islam et al,^60^ access to green spaces for mothers of lower socio-economic status would contribute to an increase in the birth weight and head circumference of the child as well as a decrease in the risk of premature birth, small stature, and atopic dermatitis.^60^ Gascon et al^59^ distinguish the effects of green spaces more generally, through four characteristics (a,b,c,d). The first characteristic (a) is residential proximity to green spaces or higher surrounding greenness, which would have an effect on physical activity, reduce childhood obesity and have a positive effect on perinatal health.^59,60^ The second characteristic (b), the presence of increased parkland, would reduce body fat in girls and, independently of obesity, lower insulin resistance in girls and boys (8-18 years), as would the third characteristic (c), outdoor surrounding greenness at home, school, and during commuting. The fourth characteristic (d), street trees, would have an effect on the prevalence of early childhood asthma, according to Johnson et al.^63^ Other studies suggest that green spaces also influence children’s respiratory and immune health overall. All the studies listed in the review by Gascon et al^59^ are yet, in the opinion of the authors, to be confirmed.

 In addition, green spaces may have a moderating effect on other determinants of children’s health. Van den Berg et al^46^ suggest that green spaces can moderate the negative impact of stressful life events on health, including three self-reported health outcomes: number of health complaints, perceived mental health, and perceived general health. Nature in the residential environment may serve as a buffer for the impact of stressful life events on rural children’s psychological well-being.^87^ Finally, urban development, especially when green spaces are sparse, combined with global warming, contributes to an increase in heat stress whose effect on health is increasingly documented.^29,41,79^ Indeed, exposure to this level of heat promotes morphological, endocrine, cardiovascular, metabolic and thermoregulatory responses that can be deleterious for children’s health and development.^80^

####  Housing

 Housing affects children’s health through two characteristics: precariousness and instability. The quality and environmental context of housing are among the main dimensions of social and environmental inequalities. Poor housing conditions are one of the mechanisms through which social and environmental inequality translates into health inequality, which further affects quality of life and well-being. According to Hunter and Flores,^52^ instability and precarious housing are associated with child abuse and neglect. The definition of housing stability varies, and includes percentage vacancy rate, rates of foreclosure and delinquency, hazardous living conditions, and instability/mobility (>1 move per year). This is partly in line with the concept of household chaos, which will be discussed below. Poor housing also exposes children to the deleterious effects of noise, especially at night,^59^ and to harmful contaminants such as tobacco smoke, carbon monoxide, lead and others.^57^ Noise, for example, will impact the child’s health in several ways (impaired reading, impaired memory, stress, several short-term physiological changes, in particular to blood pressure).^57^ Housing structure and design will also have an impact on domestic accidents depending on its structural danger (presence of windows, stairs, swimming pools, elements likely to cause burns), but also by the presence of elements available to the child such as chemical products, weapons or simply screens.^57^

####  Neighborhood social and spatial configuration

 At the neighborhood level the influences are numerous but can be distinguished through two dimensions: i) the psychosocial factors such as social cohesion, social capital and sense of community, and ii) the physical characteristics and essential services.

####  i. Socio-economic profile and network

 In almost all research, neighborhood quality is linked with the socio-economic profile of the population.^32^ Pillas et al^2^ show the negative effect of certain neighborhood-level social factors characterized by: a) area-wide unemployment levels, b) representation of lower social classes, c) low household income or d) lack of a car. Maggi et al^13^ highlight the interrelationship between family and neighborhood. On the one hand, the family characteristics buffer the neighborhood effects of school-readiness more for toddlers than for older children. On the other hand, the characteristics of the neighborhoods, notably neighborhood safety, cohesion, and crowding are a few of the factors that may inﬂuence family practices, family psychological well-being, and thus children’s development.^13^ Indeed, despite an insecure physical environment, social networks within the neighborhood can provide supportive spaces where families and children feel safe. This local social cohesion also plays an important role in children’s developmental health: learning language, managing emotions and behavior, etc.^32^ Finally, these neighborhood characteristics are directly correlated with the social support provided to mothers and the type and quality of childcare, which are particularly influential on the cognitive development of children.^56^ These social and educational support, formal or informal, would therefore be a major challenge for public investment, regardless of the profile of inhabitants.^56^

####  ii. Physical characteristics and access to essential resources

 These characteristics include (a) the density of residence and population, (b) walkability, and (c) access to essentials resources including food resources, such as open green spaces, grocery stores, schools and hospitals, and play spaces.

 Living in densely built-up areas (a) is associated with more physical activity and less sedentary time, and densely populated areas is associated with less physical activity outside school hours and more sedentary time.^61,72^ Living in a low socio-economic environment may contribute to the risk of childhood obesity through psychosocial stress and also, potentially, through reduced opportunities for physical activity due to safety and walkability issues in the neighborhood.^61^

 Walkability (b) takes into account population density, street connectivity, wealth of facilities and land use.^72^ According to Gascon et al,^59^ walkability would have an impact on physical activity behavior in a nuanced and complex way: (i) In low socio-economic neighborhoods, walkability is positively related to increased walking for transportation in leisure time, but negatively related to sport participation, (ii) areas with more traffic load are perceived as unsafe, and consequently act as barriers and discourage physical activity.^58^ Traffic load would also have a negative effect on sleep duration.^58^ All these characteristics add up negatively. As Fernández-Barrés et al^58^ point out, “Poorer areas with polluted roads and low walkability, poor quality green spaces to play and exercise and unsafe neighborhoods with low quality physical infrastructure are wider determinants that create a clustering of environmental risks that may lead to unhealthy behaviors and subsequent risk of non-communicable diseases and widening of health inequalities”. Thus, more vegetation, more building and facility density, less population density and greater distance from major roads may be associated with health-promoting behaviors in childhood.^59^

 Regarding food supply (c), Gascon et al^59^ report the results of several studies. One study shows that the density of unhealthy food outlets in a neighborhood is associated with a higher prevalence of overweight and obesity in children aged 4-11 years. A second study shows an association between greater access to fast food outlets and lower bone mineral density at age 4 years in infants, and the reverse is true if the neighborhood has more specialized shops selling healthier products. The natural playground (c)^37^ is a natural source of play, is outdoors, unstructured, fun, and intrinsically motivated. Outdoor play consists of a combination of sedentary, light, moderate, and vigorous activities. According to Herrington and Brussoni,^37^ playing outdoors, particularly in natural play spaces, stimulates children’s physical activity, which may reduce childhood obesity and allow for more varied forms of play for children of different abilities and ages. This would help to reduce inequalities, while spaces specifically structured for sport work less well for less active children. These authors also go further, proposing an evidence-based “theory of play affordances” describing 7 Cs for the design of child-friendly play spaces:

 Character (i.e., the overall feel of outdoor play spaces including light quality, color differentials, presence of soft material);

 Context (i.e., involving how the play space interacts with its surroundings);

 Connectivity (i.e., physical and visual connectedness of the play space, linked to physical and cognitive development);

 Change (i.e., referring to the range of differently sized spaces designed in the play area and how these spaces change over time, linked to cognitive and emotional development);

 Chance (i.e., providing an opportunity for children to create, manipulate, and leave an impression on their outdoor play space, accommodating spontaneous exploration, which links physical and cognitive development by prompting children to explore and discover);

 Clarity (i.e., integrating physical and perceptual legibility: play spaces should create enough mystery to promote spontaneous exploration, but not confusion that will prevent children from investigating the play space), and

 Challenge (i.e., the available physical and cognitive challenges that a play space provides, as these help children learn about their own potential, how to navigate the environment, and manage risks in other settings).^37^

####  Contaminants 

 Exposure to harmful contaminants in the physical environment is ubiquitous. According to the World Health Organization, approximately 92% of the world’s population is exposed to harmful levels of ambient air pollution.^88^ Chemicals of concern in the physical environment (i.e., soil, air, water) come from both natural and man-made sources. Regulations have been established for some, but not all, chemicals of concern because of their deleterious effects on human health, including those related to child development. In our review, 11 articles address this issue of contaminants.^40,42,61–69^

 Environmental contaminants include heavy metals such as lead, mercury, cadmium and arsenic, and persistent organic pollutants (e.g., polyfluoroalkyl substances and bisphenol A).^48,68^ These contaminants have a negative impact in both developmental (e.g., cognitive and executive functions, behavior) and physical (e.g., endocrine disruption, risk of obesity, changes in brain structure, cancers, etc.) terms. The review shows that the most documented associations between contaminants and children’s cognitive development (i.e., the acquisition of abilities such as memory, attention, reasoning and planning) concern i) children’s exposure to lead,^50,56^ or the mother’s exposure during pregnancy,^50^ ii) potential Indoor Nitrogen Dioxide exposure,^69^ iii) prenatal polycyclic aromatic hydrocarbons exposure,^69^ iv) postnatal fluoride exposure, and v) mercury exposure.

 In addition, authors point out in general that exposure to contaminants and their effects on health are complex because of the genetic vulnerability of each individual,^50^ the timing of the exposure, and combinations of multiple exposures. There is thus a real disparity in exposure, to the detriment of marginalized communities who are overexposed.^69^ Finally, there is a general consensus that children are generally more vulnerable to housing-related pollutants because they spend proportionately more time indoors than adults and are more exposed due to physiological factors (underdevelopment of their natural defense mechanisms (e.g., blood-brain barrier, immune system)) and behavioral factors, such as hand-to-mouth activity, and less awareness of the conditions in their environment.^40^ Levels of pesticides and lead in the air and in settled dust, for example, have also been found to be higher closer to the ground, increasing children’s exposure. Finally, children have a larger surface area relative to their total body mass and higher respiratory rates, and they drink more water and consume more food, thus contributing to increased exposure, especially during “critical exposure windows”.^57^

 Owino and al. explore the role of food and environmental contaminants in child growth and on the implications for metabolic dysfunction and disease risk in later life. Food contaminants can lead to growth disturbances (retarded or rapid height gain).^42^ Endocrine disruptors are associated with increased fasting insulin, increased body mass index and reduced cognitive and neurological development.^42^

 Contaminants can also be present in everyday products, for example per- or polyﬂuoroalkyl substances present in numerous products such as food packaging material, cookware, clothing, carpets, and ﬁre extinguishers.^89^ Early-life exposure to polyfluoroalkyl substances during critical periods of development may affect fetal and postnatal growth, adiposity, and pubertal development, potentially leading to latent health effects in adulthood.^64^

 In addition, exposures to these contaminants can be combined. The concept of exposome qualifies the set of environmental exposures and their interrelationships and explains both vulnerability and resilience to early neurotoxins eﬀects.^90^ It has become increasingly recognized that social and environmental risks do not exist in isolation and instead commonly co-occur, particularly among health disparity populations.^61^ Finally, risks can be moderated by psychological, biological and nutritional factors.^68^

 Air pollution refers to “a multifaceted environmental toxicant comprising a diverse mixture of particulate matter, metals, organic compounds, gases, and other chemicals (e.g., organic volatile compounds) found in the air”.^40^ Air pollution impairs health through both biological (oxidative stress, inflammation and/or endocrine disruption) and developmental (e.g. fetal growth restriction, preterm birth) effects.^48^ More specifically, air pollution has a direct or indirect impact (through maternal exposure) on asthma-related symptoms, respiratory infections, reduced lung function and obesity in children^59^ and development.^48^ Furthermore, if mothers are exposed during her pregnancy to “higher levels of air pollutants”, there is an increase in premature births and growth retardation.^63^ These results are, however, qualified by Zheng et al,^69^ who point out that “Although there is clear biological plausibility linking prenatal low-level environmental exposures to impaired fetal development and childhood growth, the epidemiologic results so far have been inconsistent. Suggestive associations are apparent, but the inconsistencies dictate further study before any of these relationships can be viewed as established”. Thus, there are numerous gaps in knowledge about the relationship between prenatal environmental exposures and fetal and early childhood growth.

 Finally, exposure disparities take other forms and affect other vulnerable populations around the world. For example, air pollution inequalities in four French metropolitan areas have been associated with the spatial and socioeconomic composition of the cities and their historical evolutions.^2^ According to Ha, the effect is moderated by socio-economic status, that is, people in lower positions are exposed to a disproportionate burden.^40^ In addition, individuals and populations with lower socio-economic status may also face other risk factors such as stress and lack of resources, which may act in synergy with air pollution to influence the risk of developmental complications. Thus, the effect of air pollution on individuals is a combination of the level of exposure on the one hand and the resources available to cope with it on the other (co-existence of other stressors).^61^ The growing recognition and evidence that air pollution is a risk factor for developmental health suggest that it may be contributing to the global increase in developmental disorders.^40^

####  The parenting environment

 While we did not plan to deal with the exercise of parenthood, two articles emerged because they treated parenthood as a social environment.^70,71^ The parenting environment acts through 4 dimensions: i) family structure (i.e., single parenthood, siblings, stability of family relationships, etc.), ii) the parents’ lifestyle behaviors (i.e., alcohol, smoking, physical activity, etc), iii) parenting behaviors (notably educational involvement and household chaos), iv) characteristics of the mother (maternal occupational class, maternal intelligence) and her behavior during and after pregnancy (i.e., risk behaviors, diet, breastfeeding). Del Carmen Ruiz et al show that all these dimensions have an impact on the child’s cognitive development.^56^ In particular, there is strong evidence that the maternal occupational class, maternal intelligence, the duration and exclusivity of breastfeeding and parenting behaviors, notably early supportive behavior and cognitive stimulation, all have an impact.^56^ In addition, the authors point out that interactions that stimulate cognitive development may serve as a protective measure against other exposures that may be negatively associated with child development. For example, social support has a protective effect on children born to mothers with high levels of stress.^56^ On the other hand, negative social interactions may exert a negative influence in themselves or reinforce the negative effects of other variables.^56^

 Andrews et al^70^ closely examine household chaos, defined as “systems of frenetic activity, lack of structure, unpredictability in everyday activities, and high levels of ambient stimulation”. This chaos is characterized by two main features, disorganization and instability. Indications of disorganization include clutter, ambient noise, overcrowding and lack of structure and routine in activities, thus evoking characteristics intrinsic to the dwelling. Instability refers to frequent changes of residence or residents (see housing instability above). In particular, these authors show that this chaos has deleterious effects on child executive functioning and effortful control. Executive functions are the essential self-regulating skills that we all use every day to accomplish just about everything. They help us to plan, organize, make decisions, move from one situation or thought to another, control our emotions and impulsiveness, and learn from past mistakes. Children use these functions for everything from showering to packing a backpack to choosing priorities. The results of Kroenke study support this by showing that the amount of parent-child speech is negatively correlated with residential crowding.^76^

 Finally, Breton et al^71^ point to an additional issue related to the family environment: epigenetic regulation of environmental inﬂuences on child health across generations. Their review identifies studies on inter- and trans-generational asthma risk related to cigarette smoke exposure. The authors also present evidence suggesting that birth weight may be related to intergenerational social determinants such as education, geographic location, and other socio-demographic characteristics. For example, grandparents’ education level and living environment have been associated with grandchildren’s birth weight. The epigenetic process suggests that the social and physical environment “gets under the skin” and marks the genetics of individuals across generations. In addition to shared genetics, families tend to have shared living environments and factors such as socio-economic status, diet, smoking and drinking, and other cultural behaviors. The family environment therefore appears to impact the health of the child through many mechanisms.

####  Social conditions

 Eleven articles specifically address this subject.^2,43–45,72–78^ Within the review carried out, three interdependent aspects are particularly identified as having a negative impact on children’s health: i) poverty, ii) low socio-economic status (economic insecurity, level of education), and iii) food insecurity.

 Indeed, poverty (i.e economic insecurity) can lead to the inability to feed, clothe, or house a child, which are basic securities for the child. According to Haft and Hoeft,^74^ poverty also has a negative impact on the development of executive functions, impairing learning to read and school performance, which are recognized as social determinants of the health of the child and the future adult. Nevertheless, these authors point out that cognitive stimulation and environmental enrichment (i.e., access to stimulating materials in the household) are variables that can moderate this effect of poverty.^74^

 Other authors have looked at the effect of economic status (ii), which is broader than economic insecurity and defined as one or a combination of measures for income (i.e., ratio of income to needs; material wealth involving family income), education (often an assessment of the mother’s highest level of education), and occupation (usually ranging from menial workers to higher executive positions). Socio-economic status has an effect on physical and psychosocial health, mediated by neighborhood structural, and behavioral and psychosocial factors (parental behavior and attitudes).^2,43^ This mediating role is also present in the work of Hunter and Flores on neglect.^52^ Finally, Schibli et al^44^ show that child poverty and low socio-economic status affect selective attention, learning and anxiety behaviors through various factors: a) stressors, especially related to chaotic environments, b) social isolation, and c) deprivation. Pillas et al^2^ characterize what they call the most significant household-level social factors: parental social class, income, employment, education (maternal and paternal), housing tenure (not owning a home) and material deprivation. Regarding parental employment, they point to differences between studies.^2^ Duncan et al^72^ explore three different avenues to explain why poverty/low socio-economic status can affect child development: the family and environmental stress perspective, the resources and investment perspective, and the cultural perspective. The first suggests that economically disadvantaged families experience more stressors in their everyday environments than do more afﬂuent families, and these disparities may affect children’s development.^72,91^ The second suggests that time and money are the two basic resources that parents use when investing in their children (investments in high-quality childcare and education, housing in good neighborhoods, and rich learning experiences enhance children’s development, as do parents’ time investments). However, the difficulties faced by the poorest families deplete their cognitive resources, increasing the likelihood that subsequent decisions will favor more impulsive and counterproductive choices. The third, the cultural perspective, follows on from the previous one. Social class differences in the parenting practices of families studied by Lareau stem,^92^ in part, from income differences that allow some to support a much wider range of activities for their children.^72^ These three different avenues can be complemented by the hypotheses of Weitzman et al, who explore the link between the socio-economic status of families and child health through access to care and the care system.^57^

 Finally, food insecurity (iii) is also reported as an important determinant. According to Hunter and Flores,^52^ there is a relationship between food insecurity and child abuse, in particular a higher exposure to parental aggression, beyond the failure to provide the basic need for food (neglect). More broadly, food insecurity is correlated with behavioral, academic and emotional problems.^78^ This is true from early childhood onwards, although these correlations differ by age. For infants and toddlers, food insecurity exposes children to problems in their physical, mental and cognitive development and in terms of attachment.^78^ In preschool children, studies have shown an association between food insecurity and externalizing behaviors (i.e., maladaptive behavior directed at an individual’s environment) and internalizing behaviors (i.e., an over-controlled and self-directed type of behavior.), mental health problems, and suboptimal development of interpersonal and self-control skills.^78^ Finally, in school-aged children, food insecurity is associated with reduced school performance, increased hyperactivity, inattention, aggressive behavior, truancy, borderline emotional issues, problems with interpersonal relationships and self-control, less adaptive approaches to learning, more internalizing and externalizing behaviors, and a greater likelihood of seeing a psychologist.^78^ Some studies go so far as to show a dose-response relationship between the duration of the experience or the level of food insecurity and school performance, use of mental health services, grade repetition and use of special education services.^78^

 This question of the effect of precarious conditions is therefore an extremely complex one. As Hackman & Farah points out,^73^ the explanation for this relationship between poverty and child health relies on a combination of genetic, epigenetic and environmental mechanisms acting across multiple factors. In particular, they open up promising causal pathways through lead exposure, cognitive stimulation including parenting behaviors, differences in the quality and quantity of schooling, nutrition, parenting styles, and transient or chronic effects of the hierarchy or stress.^73^ According to Wright,^47^ marginalized populations of lower socio-economic status are disproportionately exposed to irritants (e.g., tobacco smoke), pollutants (e.g., diesel particulate matter) and indoor allergens and may also live in more socially deleterious communities, increasing psychosocial stress which in turn, in a vicious circle, makes them more vulnerable to the full range of determinants. An illustration of this interdependence is provided by Onnis et al,^55^ who show the synergy for language development of basic cognitive mechanisms enabling learning and a rich social context from which learning takes off. Ursache and Noble suggest that socio-economic disparities in language exposure and stress may explain the association between socio-economic status and neurobiological functions for certain domains such as language, executive functions, and memory.^45^ Kroenke explores evidence that socioeconomic adversity may inﬂuence social interaction between parents and children.^76^ For example, parents of higher socio-economic status engage children more in conversations and these are more complex and involve more efforts to elicit child speech.^93^ The kinds of cognitive and psychosocial differences in children affect health over the long term. Thus, the condition of precariousness not only acts on the health and development of the child specifically through a number of mechanisms, but also constitutes a potentiator of all the other determinants.^47^

####  Climate change

 Four articles deal with the effect of climate change on children.^29,41,79,80^ Climate change impacts, which include rising temperatures, extreme weather, rising sea levels, and increasing carbon dioxide levels, are associated with a wide range of health issues in children. Indeed, children are more vulnerable because their ability to protect themselves from environmental stresses depends largely on the resources of their caregivers and the local community. In addition, their physical and psychological immaturity increases both the risk of morbidity and the risk of serious illness or lasting disability from these assaults along the life course.^94^ The health impacts of climate change are direct or indirect. Direct effects are immediate, while indirect effects are the impacts that climate change will have on important social determinants of children’s health. McMichael discusses the diffuse or “tertiary” effects of climate change,^95^ which are downstream effects such as physical displacement, altered recreational facilities and limited opportunities for children.

 Climate change acts in several ways.^96^ It increases rainfall and sea levels, coupled with ﬂooding that can lead to mold in homes, and influences air quality with direct effects (e.g., asthma) or indirect effects via air pollution. Climate change has major consequences for changes in nutrition and food security, food production systems, food safety and safe drinking water.^96^ Moreover, all these consequences are socially differentiated.^96^ They exacerbate health inequalities within countries (education, socio-economic position),^29^ between countries (high and low income), but also geographical and intergenerational inequalities. Climate change is therefore an amplifier of existing inequalities.^29,41,79,80^ By increasing the conflicts from climate-related social and political instability, displacement and forced migration, environmental refugees and disruptions in education,^94^ climate change creates new insecurities or conditions of insecurity such as national or international conflicts.^75^

###  Towards a pragmatic framework

**Figure 2:**
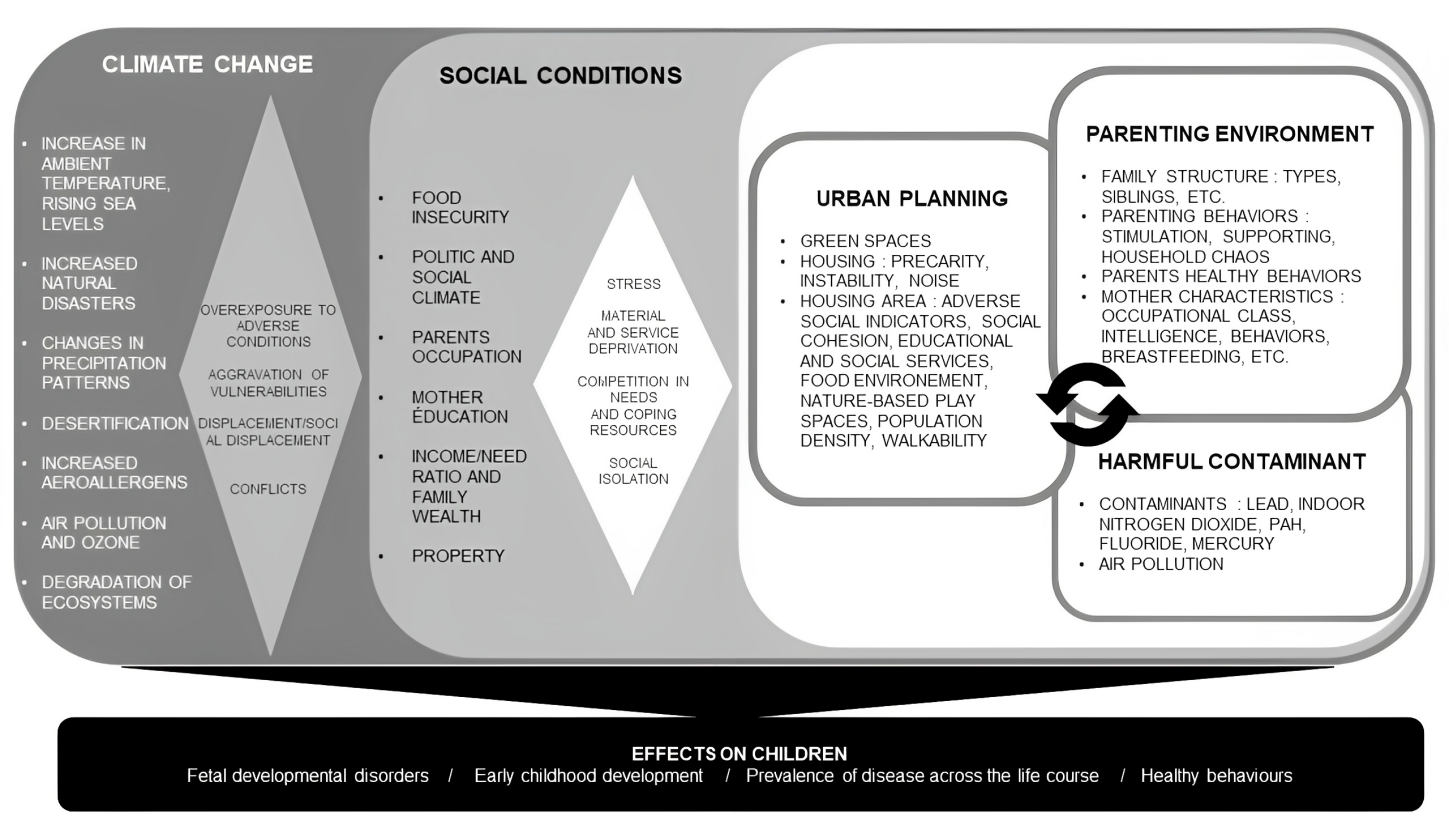
A summary figure of the effects of environmental determinants on child health. Legend: In this framework, the determinants and their characteristics are represented in the white, medium and dark gray squares. The mechanisms of action between the determinants are represented by diamonds. Together and in a systemic way, they act on the health of the child. These effects are shown in the black square

 We have identified 5 categories of determinants of the world around the child involved in child health: urban design, contaminants, the parenting environment, social conditions, and climate change. Some of their determinants act directly (exposure to a pollutant that alters brain development), others indirectly (cramped housing that causes stress, leading to less stimulation for the child and less cognitive development). Some act alone (poverty leading to a lower quality of food supply, and thus nutritional behaviors less favorable to health and development), while most act in a systemic way (climate change potentiating conditions of precariousness, neighborhood characteristics influencing parental practices which themselves influence the social characteristics of the neighborhood). It is therefore difficult to determine any form of hierarchy between them or to find any exhaustiveness in the mechanistic correlation between them and the health and well-being of children. However, the review carried out allows us at this stage to propose a summary figure for clarifying the effects of the physical, social, and economic environment on children’s health and well-being (see Figure 2).

## Discussion

 Our review aimed to clarify the environmental determinants that constitute the world around the child.^19^ As a result, we identified five categories of determinants, an outline of their interactions, and the effects of these on the health of the child. The framework proposed in Figure 2 is a synthesis of the results of the review.

 The first lesson of this review is the deeply systemic nature of the data collected. Indeed, we have seen that these determinants respond to each other and are interwoven in the process of effect. For example, urban planning influences social cohesion within neighborhoods, which influence parenting behaviors. Housing characteristics influence both parenting behaviors and exposure to contaminants (materials used). Minh et al^32^ show that the quality, structure and nature of the family environment explain at least part of the relationship between neighborhood socio-economic status and developmental health. In this sense, Robinson et al propose an urban exposome translating the correlation between exposures to the urban environment,^97^ questioning the value of isolating the analyses of each exposure. From a pragmatic point of view, the challenge is therefore to consider a global approach rather than prioritizing one element over another, as is often done in public health, in order to make cities healthier and more livable.^98^

 The second lesson is that the mechanisms through which these interactions occur are multiple, and are sometimes explored, sometimes not, sometimes simply identified by authors as a research issue. For example, we have seen that the mechanisms of effect of natural-based play are well documented. The conditions for cognitive stimulation, identified as a determinant of the parenting environment, are less so and call for further investigation: what type of stimulation, how, at what pace, based on what educational approaches? If we take the example of green spaces and their impact on physical, mental and social health, there are certainly models such as that of Le Gall et al^99^ describing macro-mechanisms of action, or comprehensive reviews such as those conducted by the EKLIPSE Expert Working Group on Biodiversity and Mental Health^100^ that detail the mental health effects of each type of green space. These models and works help to provide an explanation, but they are not specific to children’s characteristics. However, we have seen that they could qualify effects due to physical characteristics (e.g., effects of contaminants) or aspects specific to their vulnerability, such as being dependent on adults. Nevertheless, some works, not included in our review, address this mechanistic issue. For example, Alderton et al^101^ propose a mediation analysis between neighborhood characteristics on the one hand and children’s mental health on the other, thus showing the causal pathway through which the determinant acts. This type of analysis is essential from a pragmatic point of view, as the clarification of this direct or indirect action process paves the way to a whole host of specific measures, whether anticipatory (e.g., in the insulation of housing), reinforcing (e.g., in access to educational infrastructures) or compensatory (e.g., through specific initiatives to support parenthood within the neighborhood). Conversely, a failure to explain correlations can lead to interventional shortcuts or dead ends. For example, Anderko et al^79^ report that asthma and school absenteeism are closely related. Without clarification, it is difficult to know how to intervene.

 The third lesson is that little account is taken of the social gradient in the nuancing or explanation of effects. Some articles address it, for example the effect of walkability, which is socially differentiated.^59^ Johnson et al^63^ highlight the fact that racial minority and low socio-economic status groups experience a disproportionate exposure to air pollution. Coley et al^102^ point out that low-income children in particular have less access to books and computers. There is a literature in economics that seeks to assess the (causal) effects of family income on child health (without necessarily identifying mechanisms). The following review reports a number of results from the last 20 years on the subject.^103^ However, not all the articles explore this aspect and most of them do so without nuancing through a social gradient. This is all the more important as health-in-all-policies (i.e., systematically taking into account health considerations in non-health policy) is an approach that aims primarily at health equity.^104^ Thus, some work has focused on identifying ways to compensate for precariousness. The Baby First Years Cohort evaluates the effect of an unconditional financial gift during the first 52 months of a child’s life on the infant’s brain development.^105^ Others investigate measures that impact the mediating determinants of precariousness, such as access to quality childcare facilities.^106^ Others could address even more structural measures or mechanisms of inequality, such as social position.^107^ Here, what might seem intractable, such as eradicating precariousness, can be addressed through a combination of interventions that tackle the effects of this structural determinant.^101^

 All of these lessons, as well as our attempt to map the different determinants to which children are exposed, reinforce awareness of the vulnerability of children. Environmental inequalities in health are characterized by vulnerability differentials and exposure differentials The exposome is a measure of all the exposures of an individual across the life course and the relationship of those exposures to health effects.^108^ It makes it possible to characterize the risk and protective factors, the mediating or moderating elements and their links with health. Juarez et al^109^ take this concept and apply it more broadly to public health: the “public health exposome”. Along these lines, this proposed mapping could be a first attempt to clarify the “childhood exposome”, understood as the sum total of exposures to which the child is “subjected”. And this notion of submission is not trivial, because the environments are designed by adults, leading to mistakes.^110,111^ As Fernández-Barrés et al point out, while land use mix is related to greater physical activity in adults, the results are mixed for children.^58^ Similarly, Herrigton and Brussoni point out that a slide designed to be used in only one way (sliding in a seated position) reassures the parent, but does not meet the 7 criteria of play affordances^37^: the child cannot climb, manipulate, invent with a slide. Also, parents’ concerns about child safety may affect a child’s ability to use a play area in a variety of ways, thereby reducing their experiences.^13^ This calls for a more systematic involvement of children in the design of facilities for them to identify how they experience things rather than relying on an experience projected by adults.

 Finally, the selection of articles, although focused on the child’s environment, identified works relating to parental behaviour. However, the analysis of the articles clearly shows that they are themselves influenced by the other determinants (particularly urban planning and social conditions) and cannot be considered in isolation. This is why, in Figure 2, we have placed them in close connection with the determinants linked to the living environment and under the direct influence of social conditions.

## Limitations

 Our review is not exhaustive. Some articles, however interesting, were not found, even though they were identified in our exploratory research or were cited in the references of the selected articles. This is unfortunate because some references can contradict or shades the results obtained in this review. For instance, Zou et al^112^ show that higher population density encourages more active behaviors, thus contradicting the work of Fernández-Barrés et al.^58^ Of course, there are many controversies in science. This further confirms the value of mechanistic explanations that allow us to explore the conditions under which elements work together to create or not create health or health equity.

 The determinants identified through our review do not have the same effects depending on the context in which they are found. For example, climate change will not have the same effects in different countries (depending on their income level, location, protection policies, etc). We have highlighted the difficulty of identifying the mechanisms by which the determinants act on the health of the child. These mechanisms are complex and differ according to the context, whether it is the level of wealth of the country or region, the policies in place or cultural elements (e.g. the relationship with nature, mutual aid within communities). The contexts and mechanisms have been poorly described in the included reviews. We regret this as it is a major limitation of our work. Furthermore, we did not include in our review determinants specific to least developed countries such as access to water and sanitation. These determinants and the specific context of these countries would have called for specific challenges and solutions. These issues would deserve an in-depth research of their own.

 In addition, we have identified a lack of data on children’s mental health. Physical health (by pathology) and development are present in most of the literature. This observation, as well as the call to investigate this outcome more widely, is congruent with the UNICEF report^19^ on the trajectory of mental capital through life^113^ or Allen and colleagues’ works^114^ that synthesize the social determinants of children’s mental health. Finally, we only searched in the PubMed database, assuming that it was the one most likely to provide information on environmental aspects. However, the fact that we collected articles on parenting environment leads us to think that in this field, we could have looked for more articles in databases more oriented towards human and social sciences, such as Scopus or Web of Science.

## Conclusion

 Our review aimed to clarify the main environmental determinants impacting children’s health. It identified five categories of interrelated determinants. It allowed us to propose a model designed as a reading grid for public policies. We were not able to identify all the mechanisms of action on health, whether direct or indirect, but the proposed model nevertheless constitutes a concrete basis for global reflection on Health in All policies dedicated to children. But the science of problems, as Louise Potvin calls it, does not produce “solutions”.^115^ How to promote play spaces that meet the 7 criteria of the 7CS model of play affordance?^37^ How can we ensure that the characteristics of a neighborhood are child-friendly when developing it? What level of street connectivity promotes access to services, social mix, but also safety and serenity in a neighborhood? How should green spaces be distributed throughout the city? What neighborhood characteristics should be adopted to reduce the effects of precariousness? These questions fall under the heading of “solution science”.^115^ Indeed, although there are many recommendations to promote the well-being of the population in the city, in neighborhoods and in organizations, they remain general and are often aimed at adults without considering the specificities and experience of children. This review thus opens up many research avenues. Finally, our question is linked to the issue of knowledge transfer. The use of science in decision-making is faced with many conditions related to data, actors and organizations.^116–121^ A commitment by all actors, researchers and decision-makers to consultation and collaboration is the sine qua non for more child-friendly environments.

## Acknowledgments

 We are Grateful to the steering committee, the living lab and the funders of the APPIE project.

## Competing Interests

 The authors declare that they have no financial or non-financial relationships and activities with regard to the publication of their work.

## Ethical Approval

 Not applicable.

## Funding

 This umbrella review is part of the APPIE project (Analysis of Public Policies with Impact on Children). This project was funded by IReSP for the Call for Research Projects 2021 on Services, Interventions and Health Promoting Policies (SIP) supported by the CNAM, DGS, Inserm, MILDECA and Santé publique France (IReSP-AAP SIP 2021 – 273255) and also by Institut national du cancer (Inca_1682).

## Supplementary Files

Supplementary file 1. Description of the 47 papers included in the review (result of the PRISMA flow chart).
