# Supplementary material for: Environment and child well-being: A scoping review of reviews to guide policies
Source: Health Promot Perspect. 2023 Sep 11;13(3):168–82. doi: 10.34172/hpp.2023.20 (PMC10558968; doi:10.34172/hpp.2023.20)
Supplement: Supplementary file 1 — Description of the 47 papers included in the review (result of the PRISMA flow chart). [file hpp-13-168-s001.pdf]

Wallerich et al, **Health Promotion Perspectives**, 2023, 13(3), S1.
doi: 10.34172/hpp.2023.20
https://hpp.tbzmed.ac.ir

Supplementary file 1. Description of the 47 papers included in the review (result of the PRISMA flow chart)

References	Author	Publication Year	Titre	Type de review	Classification
**2**	Pillas, Demetris	2014	Social inequalities in early childhood health and development: a European-wide systematic review	systematic review	Social conditions
**13**	Maggi, Stefania	2010	The social determinants of early child development: An overview	Overview	Multiplicity/Interaction
**29**	Arpin, Emmanuelle	2021	Climate Change and Child Health Inequality: A Review of Reviews	A Review of Reviews	Climate change
**32**	Minh, Anita	2017	A review of neighborhood effects and early child development: How, where, and for whom, do neighborhoods matter?	review	Urban planning
**37**	Herrington, Susan	2015	Beyond Physical Activity: The Importance of Play and Nature-Based Play Spaces for Children's Health and Development	review	Urban planning
**39**	Barrett, Emily S.	2019	Joint Impact of Synthetic Chemical and Non-Chemical Stressors on Children's Health	review	Multiplicity/Interaction
**40**	Ha, Sandie	2021	Air pollution and neurological development in children	review	Contaminant
**41**	Helldén, Daniel	2021	Climate change and child health: a scoping review and an expanded conceptual framework	Scoping review	Climate change
**42**	Owino, Victor O.	2018	Elucidating Adverse Nutritional Implications of Exposure to Endocrine-Disrupting Chemicals and Mycotoxins through Stable Isotope Techniques	review	Contaminant
**43**	Poulain, Tanja	2020	Review on the role of socioeconomic status in child health and development	review	Social conditions
**44**	Schibli, Kylie	2017	Attending, learning, and socioeconomic disadvantage: developmental cognitive and social neuroscience of resilience and vulnerability	review	Social conditions
**45**	Ursache, Alexandra	2016	Neurocognitive development in socioeconomic context: multiple mechanisms and implications for measuring socioeconomic status	review	Social conditions

**46**	van den Berg, Agnes E.	2010	Green space as a buffer between stressful life events and health	review	Urban planning
**47**	Wright, Rosalind J.	2009	Moving towards making social toxins mainstream in children's environmental health	review	Multiplicity/Interaction
**48**	Abdul-Hussein, Ayah	2021	Early life risk and resiliency factors and their influences on developmental outcomes and disease pathways: a rapid evidence review of systematic reviews and meta-analyses	a rapid evidence review of systematic reviews and meta-analyses	Multiplicity/Interaction
**49**	Adegbosin, Adeyinka E.	2019	Systematic review and meta-analysis of the association between dimensions of inequality and a selection of indicators of Reproductive, Maternal, Newborn and Child Health (RMNCH)	review of systematic reviews and meta-analyses	Multiplicity/Interaction
**50**	Ferguson, Kim T.	2013	The physical environment and child development: An international review	review	Multiplicity/Interaction
**51**	Hibbert, Kathleen	2019	State-of-the-Science Review of Non-Chemical Stressors Found in a Child's Social Environment	review	Multiplicity/Interaction
**52**	Hunter, Amy A.	2021	Social determinants of health and child maltreatment: a systematic review	systematic review	Multiplicity/Interaction
**53**	Mitchell, Colter	2016	DNA methylation, early life environment, and health outcomes	review	Multiplicity/Interaction
**54**	Nelson, Helen Jean	2014	Neurological and Biological Foundations of Children's Social and Emotional Development: An Integrated Literature Review	review	Multiplicity/Interaction
**55**	Onnis, Luca	2018	Language development and disorders: Possible genes and environment interactions	review	Multiplicity/Interaction
**56**	Ruiz, Jazmin Del Carmen	2016	Contributions of a Child's Built, Natural, and Social Environments to Their General Cognitive Ability: A Systematic Scoping Review	A Systematic Scoping Review	Multiplicity/Interaction
**57**	Weitzman, Michael	2013	Housing and Child Health	review	Multiplicity/Interaction
**58**	Fernández-Barrés, Sílvia	2022	Urban environment and health behaviours in children from six European countries	Research Support	Urban planning

**59**	Gascon, Mireia	2016	The Built Environment and Child Health: An Overview of Current Evidence	review	Urban planning
**60**	Islam, Mohammad Zahirul	2020	Green space and early childhood development: a systematic review	a systematic review	Urban planning
**61**	Appleton, Allison A.	2016	A Systematic Review of the Interplay Between Social Determinants and Environmental Exposures for Early-Life Outcomes	Systematic review	Contaminant
**62**	Dórea, José G.	2019	Environmental exposure to low-level lead (Pb) co-occurring with other neurotoxicants in early life and neurodevelopment of children.	review	Contaminant
**63**	Johnson, Natalie M.	2021	Air pollution and children's health—a review of adverse effects associated with prenatal exposure from fine to ultrafine particulate matter	review	Contaminant
**64**	Lee, Yun Jeong	2021	Early-Life Exposure to Per- and PolyFluorinated Alkyl Substances and Growth, Adiposity, and Puberty in Children: A Systematic Review	A Systematic Review	Contaminant
**65**	Lin, Wei-Wei	2017	Air Pollution and Children's Health in Chinese	review	Contaminant
**66**	Mastorci, Francesca	2021	Environment in Children's Health: A New Challenge for Risk Assessment	review	Contaminant
**67**	Myhre, Oddvar	2018	Early life exposure to air pollution particulate matter (PM) as risk factor for attention deficit/hyperactivity disorder (ADHD): Need for novel strategies for mechanisms and causalities	review	Contaminant
**68**	Trentacosta, Christopher J.	2020	New Directions in Understanding the Role of Environmental Contaminants in Child Development: Four Themes	review	Contaminant
**69**	Zheng, Tongzhang	2016	Effects of Environmental Exposures on Fetal and Childhood Growth Trajectories	review	Contaminant
**70**	Andrews, Krysta	2021	Examining the Effects of Household Chaos on Child Executive Functions: A Meta-Analysis	Meta analyse	Parenting environment
**71**	Breton, Carrie V.	2021	Exploring the evidence for epigenetic regulation of environmental influences on child health across generations	review	Parenting environment
**72**	Duncan, Greg J.	2017	Moving Beyond Correlations in Assessing the Consequences of Poverty.	review	Social conditions
**73**	Hackman, Daniel A.	2009	Socioeconomic status and the developing brain	review	Social conditions
**74**	Haft, Stephanie L.	2017	Poverty's Impact on Children's Executive Functions: Global Considerations.	review	Social conditions

**75**	Kadir, Ayesha	2019	Effects of armed conflict on child health and development: A systematic review	A systematic review	Social conditions
**76**	Kroenke, Candyce	2008	Socioeconomic status and health: Youth development and neomaterialist and psychosocial mechanisms	review	Social conditions
**77**	Olson, Lindsay	2021	Neural Correlates of Socioeconomic Status in Early Childhood: A Systematic Review of the Literature	Systematic Review	Social conditions
**78**	Shankar, Priya	2017	Association of Food Insecurity with Children's Behavioral, Emotional, and Academic Outcomes: A Systematic Review	Systematic Review	Social conditions s
**79**	Anderko, Laura	2020	Climate changes reproductive and children's health: a review of risks, exposures, and impacts	review	Climate change
**80**	Smith, Caroline J.	2019	Pediatric Thermoregulation: Considerations in the Face of Global Climate Change	review	Climate change
